# Supplementary material for: Transcriptomic Profiling of Young Cotyledons Response to Chilling Stress in Two Contrasting Cotton (Gossypium hirsutum L.) Genotypes at the Seedling Stage
Source: Int J Mol Sci. 2020 Jul 19;21(14):5095. doi: 10.3390/ijms21145095 (PMC7404027; doi:10.3390/ijms21145095)
Supplement: Supplementary file 1 [file ijms-21-05095-s001.zip › Supplementary Files/Figure S1.pdf]

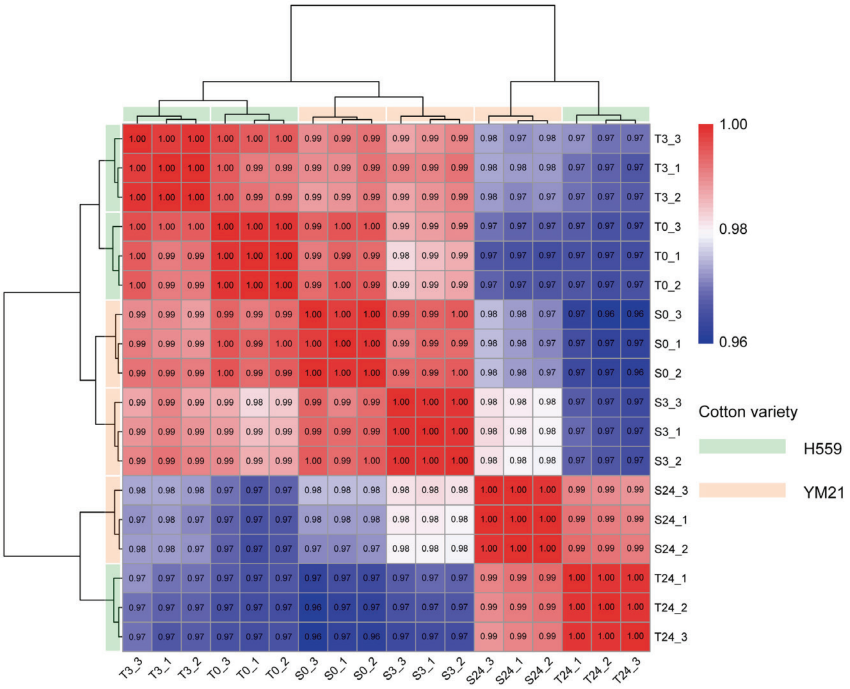

Figure S1. Pearson correlation coefficient analysis of transcriptome data from cotyledons of two cotton varieties under chilling stress. The relationship between samples was also analyzed by hierarchical clustering dendrogram.
