# Supplementary material for: Transcriptomic Profiling of Young Cotyledons Response to Chilling Stress in Two Contrasting Cotton (Gossypium hirsutum L.) Genotypes at the Seedling Stage
Source: Int J Mol Sci. 2020 Jul 19;21(14):5095. doi: 10.3390/ijms21145095 (PMC7404027; doi:10.3390/ijms21145095)
Supplement: Supplementary file 1 [file ijms-21-05095-s001.zip › Supplementary Files/Figure S2.pdf]

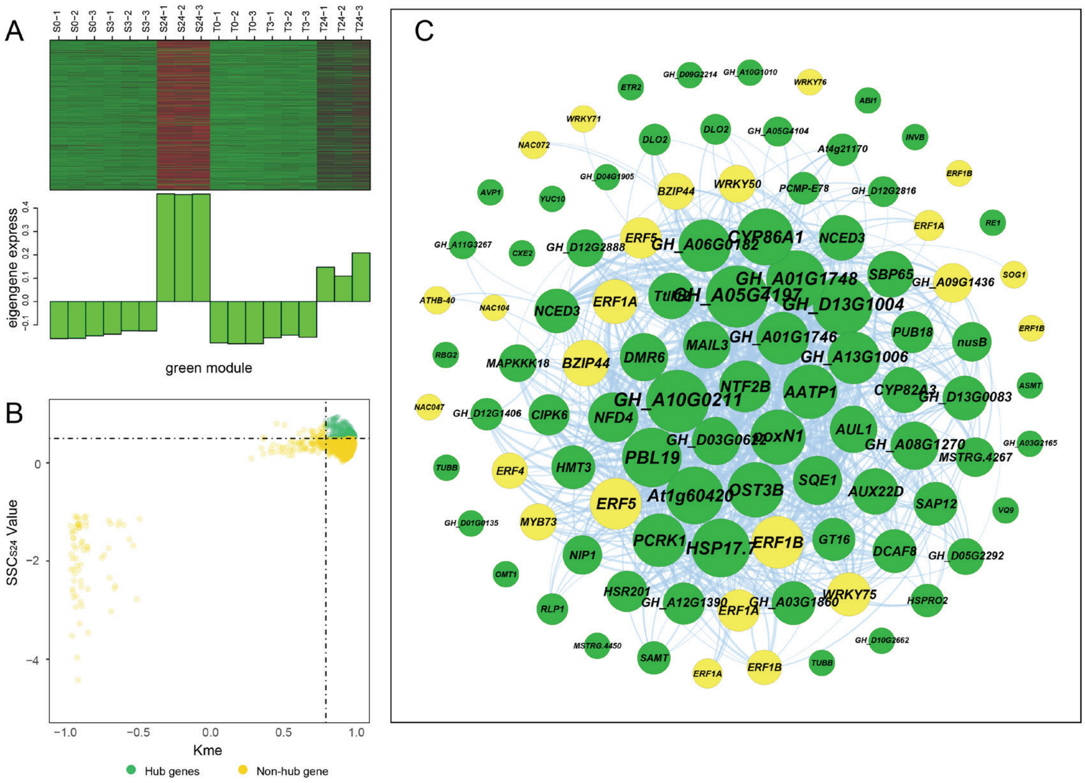

Figure S2. Co-expression network construction of the green module. (**A**) Gene co-expression heatmap of the green module (upper panel) and the expression level of the corresponding eigengene in each sample (lower panel). (**B**) Dot plot for mining hub genes with higher module connectivity and higher expression levels in S24. The X-axis represents connectivity values (Kme) of genes in the green module, while the Y-axis represents preponderant expression value (SSC_{S24}) of the genes at S24. Genes with Kme values > 0.8 and SSC_{T24} values > 0.50 were identified as hub genes (green dots). (**C**) The gene co-expression network of the green module with hub genes. The top 100 hub genes with higher weight value (> 0.35) and higher expression levels in S24 were used to construct the network. The node size indicates gene degree, and the node colour is used to distinguish whether it is a TF gene (orange node). The thickness of the line reflects weight value.
