# Supplementary material for: Transcriptomic Profiling of Young Cotyledons Response to Chilling Stress in Two Contrasting Cotton (Gossypium hirsutum L.) Genotypes at the Seedling Stage
Source: Int J Mol Sci. 2020 Jul 19;21(14):5095. doi: 10.3390/ijms21145095 (PMC7404027; doi:10.3390/ijms21145095)
Supplement: Supplementary file 1 [file ijms-21-05095-s001.zip › Supplementary Files/Figure S3.pdf]

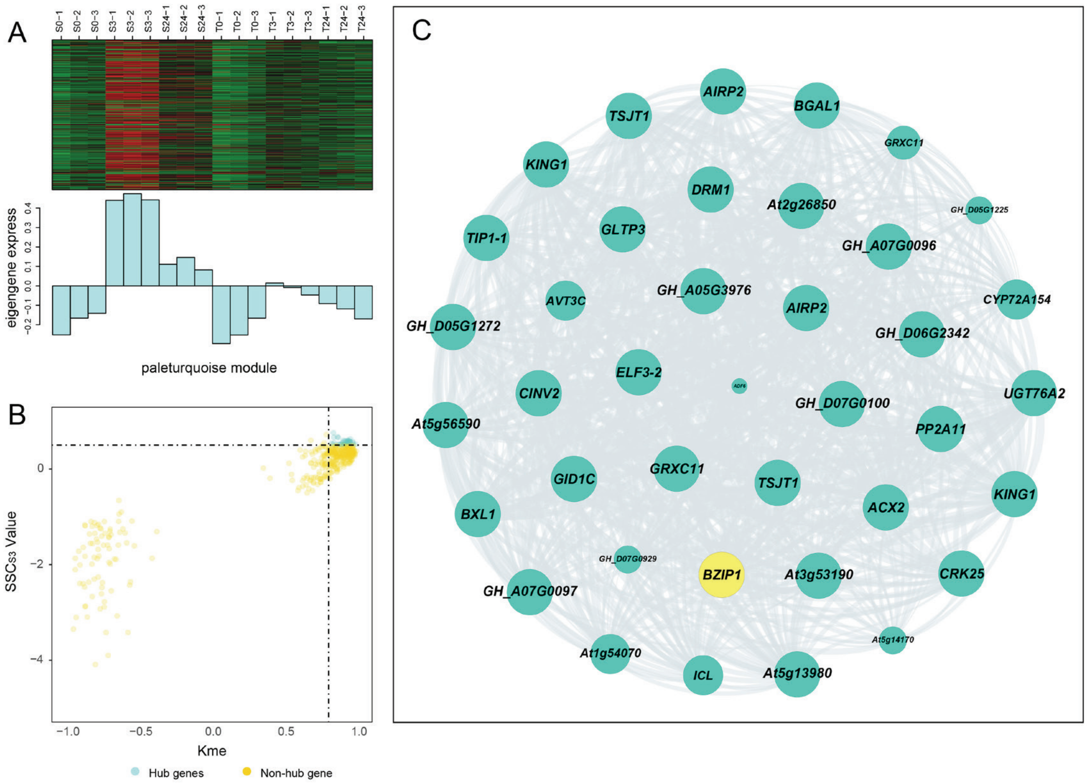

Figure S3. Gene co-expression network construction of the paleturquoise module. (A) Gene co-expression heatmap of the paleturquoise module (upper panel) and the expression level of the corresponding eigengene in each sample (lower panel). (B) Dot plot mining hub genes with higher module connectivity and higher expression levels in S3. The X-axis represents the connectivity values (Kme) of genes in the paleturquoise module, while the Y-axis represents preponderant expression value (SSC_{S3}) of the genes in S3. Genes with Kme values > 0.8 and SSC_{S3} values > 0.5 were identified as hub genes (paleturquoise dots). (C) The correlation network of the paleturquoise module with all co-expressed genes. The node size indicates gene degree. The thickness of the line reflects weight value.
